# Supplementary material for: Optimal time lags from causal prediction model help stratify and forecast nervous system pathology
Source: Sci Rep. 2021 Oct 22;11:20904. doi: 10.1038/s41598-021-00156-2 (PMC8536772; doi:10.1038/s41598-021-00156-2)
Supplement: Supplementary file 1 — Supplementary Information. [file 41598_2021_156_MOESM1_ESM.docx]

**Supplementary Table 1: Summary Demographics (All groups)**

|  | **Mean Age** | **Females (Total)** | **Males (Total)** |
| --- | --- | --- | --- |
| **Young Controls** | 10 (+/- 4) | 4 | 4 |
| **College Aged Controls** | 19 (+/- 2) | 13 | 2 |
| **Adult Controls** | 29 (+/- 4) | 6 | 2 |
| **Elderly Controls** | 58 (+/- 13) | 3 | 1 |
| **Tourette’s ASD** | 8 | - | 1 |
| **Idiopathic ASD** | 8 (+/- 3) | 1 | 3 |
| **Fragile X Carriers** | 37 (+/- 21) | 6 | 1 |
| **Fragile X Full Mutation** | 13 | - | 1 |
| **DHI Participants** | 73 (+/- 8) | 61 | 57 |
| **SHANK3 Participants** | 8 (+/- 3) | 8 | 7 |
| **Parkinsonian Participants** | 69 (+/- 5) | 3 | 4 |
| **Total (188)** |  | 106 | 82 |

**Supplementary Table 2: Summary Demographics DHI Group**

| **N=118** | |
| --- | --- |
| **Age mean (SD)** | 73.25 (8.38) |
| **Sex n (%)**  Male  Female | 57 (48.3)  61 (51.7) |
| **Race/Ethn n (%)**  NH-White  Others | 66 (55.9)  52 (44.1) |
| **DHI-S mean (SD)**  Mild 0-12 n(%)  Moderate 14-24 n (%)  Severe 26-40 n (%) | 6.29 (8.77)  96 (81.4)  15 (12.7)  7 (5.9) |
| **Chief Complaints**  Hearing loss n (%)  Tinnitus n (%)  Dizziness n (%)  Others n (%) | 99 (83.9)  48 (40.7)  53 (44.9)  19 (16.1) |

DHI-S indicates dizziness handicap inventory—screening version; NH-White, non-Hispanic white; Race/Ethn, race/ethnicity; SD, standard deviation.

Several scores are depicted in Table 3 below, from the following clinical tests used to assess various motor and cognitive aspects of behavior in Autism:

[1] The Mullen Scales of Early Learning; Gross Motor and Fine Motor scores provided are the age equivalent in months

[2] ADI-R cutoff scores for autism are: Social = 10, Communication (nonverbal) = 7, Repetitive Behaviors and Restricted Interests = 3

[3] All participants received a Toddler Module or Module 1 of the ADOS-2

[4] The Mullen Scales of Early Learning; DVIQ scores were calculated by averaging the age equivalent for fine motor and visual reception subtests then dividing by the child's age in months and multiplying by 100 (Bishop, Guthrie, Coffing, & Lord, 2011)

**Supplementary Table 3: SHANK3-ASD demographic and clinical information**

| **Subject** | **Age yr:mo** | **The Mullen Scales of Early Learning [1]** | | | **Vineland Adaptive Behavior Scales-II** | | | | | **ADI-R [2]** | | | **ADOS-2 [3]** | | **DSM-IV/ DSM-5** | **Consensus Diagnosis** |
| --- | --- | --- | --- | --- | --- | --- | --- | --- | --- | --- | --- | --- | --- | --- | --- | --- |
|  |  | **Gross Motor** | **Fine Motor** | **NVDQ estimate [4]** | **Motor** | **Communication** | **Socialization** | **Daily Living** | **ABC** | **A: Soc** | **B: Com** | **C: RRBI** | **SA** | **RRB** |  |  |
| 1 | 5:11 | 18 | 14 | 17.79 | 56 | 42 | 51 | 40 | 45 | 19 | 13 | 1 | 18 | 18 | ASD | ASD |
| 2 | 8:5 | 16 | 13 | 17.81 | 54 | 38 | 48 | 48 | 44 | 23 | 14 | 6 | 19 | 19 | ASD | ASD |
| 3 | 6:4 | 27 | 28 | 21.12 | 61 | 44 | 59 | 51 | 52 | 19 | 10 | 2 | 14 | 14 | ASD | ASD |
| 4 | 5:11 | - | 22 | 27.46 | 59 | 49 | 57 | 48 | 51 | 11 | 13 | 3 | 7 | 7 | ASD | ASD |
| 5 | 6:1 | 18 | 9 | 13.69 | 54 | 36 | 48 | 34 | 41 | 28 | 14 | 7 | 17 | 17 | ASD | ASD |
| 6 | 7:10 | 20 | 15 | 15.43 | 49 | 45 | 48 | 51 | 48 | 30 | 14 | 6 | 20 | 20 | ASD | ASD |
| 7 | 7:8 | 33 | 33 | 8.68 | 56 | 45 | 53 | 51 | 50 | 23 | 13 | 6 | 16 | 16 | ASD | ASD |
| 8 | 9:1 | 13 | 15 | 17.09 | 81 | 54 | 66 | 63 | 61 | 5 | 6 | 4 | 15 | 15 | ASD | ASD |
| 9 | 14:8 | 29 | 28 | 19.39 | 56 | 35 | 32 | 35 | 31 | 16 | 12 | 8 | 20 | 20 | ASD | ASD |
| 10 | 5:11 | 22 | 10 | 9.86 | 56 | 36 | 51 | 38 | 43 | 24 | 12 | 5 | 18 | 18 | ASD | ASD |
| 11 | 15:9 | - | 6 | 4.22 | 56 | 33 | 37 | 28 | 29 | 28 | 14 | 2 | 17 | 17 | ASD | ASD |
| 12 | 5:0 | 14 | 6 | 26.67 | 43 | 40 | 51 | 41 | 43 | 25 | 14 | 8 | 4 | 14 | ASD | ASD |
| 13 | 8:7 | 15 | 13 | 12.01 | 43 | 47 | 48 | 48 | 47 | 28 | 14 | 2 | 5 | 10 | ASD | ASD |
| 14 | 5:9 | 22 | 21 | 34.06 | 54 | 61 | 61 | 55 | 55 | 13 | 15 | 0 | 16 | 4 | ASD | ASD |
| 15 | 5:1 | 21 | 17 | 36.07 | 56 | 52 | 51 | 43 | 49 | 23 | 14 | 6 | 20 | 5 | ASD | ASD |
| 16 | 12:6 | 30 | 20 | 13.51 | 64 | 42 | 42 | 50 | 44 | 29 | 13 | 4 | 22 | 3 | ASD | ASD |

Abbreviations: ABC, Adaptive Behavior Composite (Vineland); ADI-R, Autism Diagnostic Interview-Revised; A: Social, Qualitative Abnormalities in Reciprocal Social Interaction; B: Com, Communication; C: Repetitive and Restricted Behaviors; ADOS, Autism Diagnostic Observation Schedule; Com Total, ADOS-2 SA, Social Affect Total, ADOS-2 RRB, Restricted and Repetitive Behavior Total; NVDQ, Nonverbal Developmental Quotient

**Supplementary Table 4: Idiopathic ASD and Age-matched and older controls**

| **Subject** | **Chronological age**  **(years)** | **Sex** | **Subject Type** |
| --- | --- | --- | --- |
| 17 | 12 | M | Idiopathic ASD |
| 18 | 11 | M | Idiopathic ASD |
| 19 | 10 | M | Idiopathic ASD |
| 20 | 7 | F | Idiopathic ASD |
| 21 | 8 | M | Tourette’s ASD |
| NEUROTYPICAL CONTROLS | | | |
| 22 | 5 | F | Neurotypical |
| 23 | 6 | F | Neurotypical |
| 24 | 7 | F | Neurotypical |
| 25 | 8 | M | Neurotypical |
| 26 | 13 | M | Neurotypical |
| 27 | 7 | M | Neurotypical |
| 28 | 16 | F | Neurotypical |
| 29 | 19 | M | Neurotypical - Athlete |
| 30 | 18 | F | Neurotypical |
| 31 | 16 | M | Neurotypical |
| 32 | 17 | M | Neurotypical |

**Supplementary Table 5. Demographics of Patients with Parkinson’s Disease and age-matched controls**

| Participant^1^ | Gender | Age | UPDRS | Hoehn and Yahr scale | Medication (Motor/Non-Motor)^2^ |
| --- | --- | --- | --- | --- | --- |
| PPD 1 | M | 56 | 6 | 2.5 | Yes/Yes |
| PPD 2 | F | 64 | 44 | 4 | Yes/Yes |
| PPD 3 | M | 65 | 24.5 | 2.5 | Yes/Yes |
| PPD 4 | M | 70 | 19 | 3 | Yes/Yes |
| PPD 5 | M | 59 | 29 | 3 | Yes/Yes |
| PPD 6 | F | 69 | - | 3 | Yes/No |
| PPD 7 | M | 64 | 16 | 2 | Yes/Yes |
| PPD 8 | M | 76 | 21 | 3 | Yes/No |
| PPD 9 | F | 77 | 27 | 3 | Yes/Yes |
| PPD 10 | M | 50 | - | 2 | Yes/Yes |
| PPD 11 | M | 63 | 20 | - | - |
| Elderly NT 1 | F | 65 | - | - | - |
| Elderly NT 2 | M | 68 | - | - | - |
| Elderly NT 3 | F | 57 | - | - | - |
| Elderly NT 4 | F | 76 | - | - | - |
| Elderly NT 5 | M | 77 | - | - | - |

^1^Participant Abbreviation

PPD: Patient with Parkinson's Disease

Elderly Control: age matched neurotypical control

^2^ Intake of medication for motor issues / medication for non-motor issues that are known to affect motor performance

**Supplementary Table 6: Abbreviations of Body Parts**

| ^Body Part^ | ^Abbreviation of body part for FMR1/PPD dataset^ | ^Abbreviation of body part for Shank3 dataset^ |
| --- | --- | --- |
| ^Pelvis^ | ^P^ |  |
| ^Lumbar Level 5^ | ^L5^ | ^L^ |
| ^Lumbar Level 3^ | ^L3^ | ^L^ |
| ^Thoracic Level 12^ | ^T12^ | ^T^ |
| ^Thoracic Level 8^ | ^T8^ | ^T^ |
| ^Neck^ | ^N^ |  |
| ^Head^ | ^H^ | ^H^ |
| ^Right Shoulder^ | ^RSH^ | ^RSH^ |
| ^Right Upper Arm^ | ^RUA^ | ^RUPP^ |
| ^Right Forearm^ | ^RF^ | ^RFORE^ |
| ^Right Hand^ | ^RH^ |  |
| ^Left Shoulder^ | ^LSH^ | ^LSH^ |
| ^Left Upper Arm^ | ^LUA^ | ^LUPP^ |
| ^Left Forearm^ | ^LF^ | ^LFORE^ |
| ^Left Hand^ | ^LH^ |  |
| ^Right Upper Leg^ | ^RUL^ | ^RTH^ |
| ^Right Lower Leg^ | ^RLL^ | ^RSK^ |
| ^Right Foot^ | ^RFoot^ | ^RFT^ |
| ^Right Toe^ | ^RT^ |  |
| ^Left Upper Leg^ | ^LUL^ | ^LTH^ |
| ^Left Lower Leg^ | ^LLL^ | ^LSK^ |
| ^Left Foot^ | ^LFoot^ | ^LFT^ |
| ^Left Toe^ | ^LT^ |  |

**A form of Noise Cancellation with Possible Therapeutic Value**

The value of examining feedback loops, timing, and stochastic signatures across the population, can be better appreciated within the context of noise cancellation. Noise cancellation helps engineer solutions and build accommodations for PPD and other disorders of the nervous systems with tremor-related signals. Along those lines, here we investigated how removing different frequency bands from the angular speeds of the PPD affected the network dynamics. We tried different combinations of low and high limits for the frequency stop band to filter out tremor components (as reported by the literature [52](#_ENREF_52) and performed *t-tests* between the resulting group and the elderly or young control groups for each body node. Then, we measured the number of nodes in which the two groups differed significantly in the NSR. We then interpolated a surface on the parameter identification data.

We report that the optimal tremor removal when comparing to the healthy young controls group is for frequencies above 19 Hz, while the optimal tremor removal when comparing to the old controls groups is for frequency band with low limits near 10 Hz. Figure 9 shows these differences.


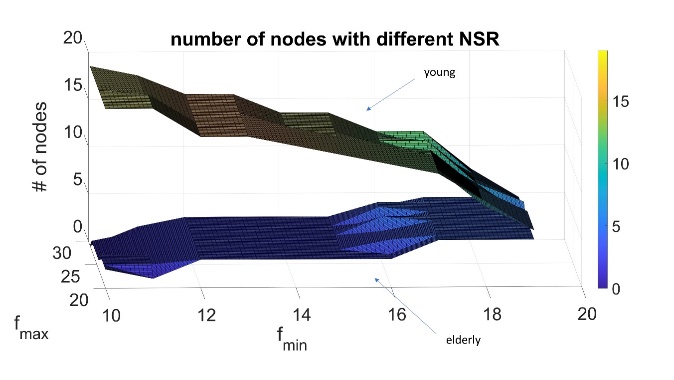


Supplementary Material Figure 1: Noise cancellation by systematic frequency removal in young *vs.* elderly participants. The two surface plots show how after removing a component of tremor from the PPD by using band stop filtering of the angular velocity for different choices of frequency, the resulting body node networks is comparable to the NSR dimension of the young and elderly controls groups. The color bar reflects the number of nodes with different NSR that results from the noise cancellation.

**MMS Amplitude and Timings are Not Independent**

Lastly, to further understand the spatio-temporal characteristics of the MMS signal derived from the angular speed, we calculated the empirical joint distributions of the MMS inter-peak-interval times and the distributions of the MMS amplitude (peak values). We asked if they were independent. The results are shown in Figure 10. The complex patterns that emerge indicate that assuming independence between the rate of MMS activity and the MMS values is only a first approximation to a more complex mechanism that needs to be further investigated.


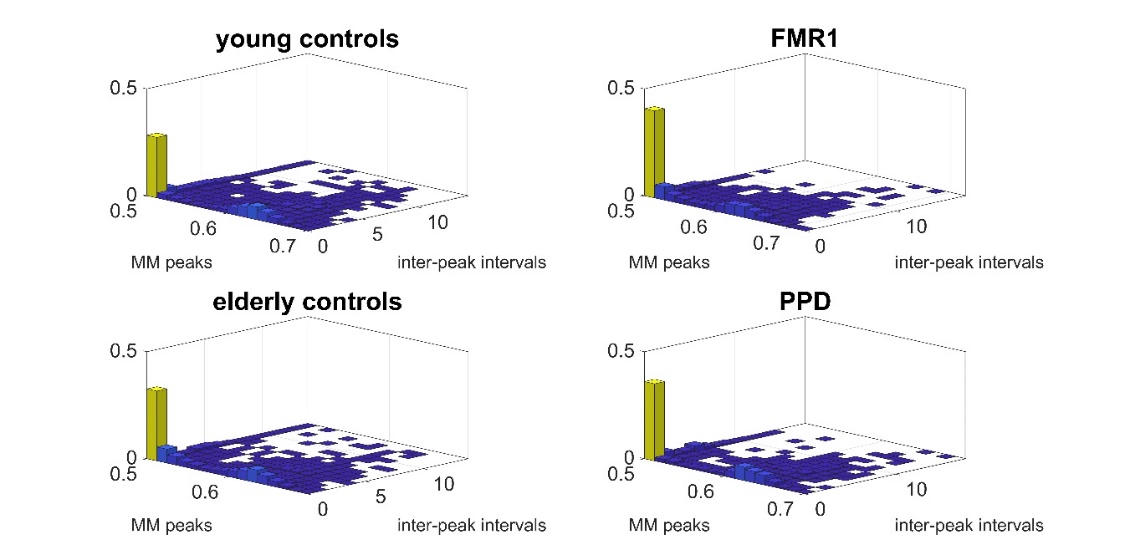


Supplementary Material Figure 2: Joint empirical distribution of MMS peak values and inter-peak-interval times.

**Significance statistical test for simplified/one-lag Granger Causality**

1. For two processes X and Y, estimate the non-causal (X 🡪 X) and causal models (Y 🡪 X) for various lags.
2. For the model that maximizes $GC=log(\frac{s_{1}^{2}}{s_{2}^{2}})$, where $s_{1}^{2}$ is the sample estimate of the variance of the non-causal model and $s_{2}^{2}$the sample estimate of the variance of the causal model for the model of that specific lag, test whether the variance $\sigma_{1}^{2}$ of the non-causal model is significantly higher that the variance $\sigma_{2}^{2}$ of the causal model.
3. The null hypothesis H_0_ : $\sigma_{2}^{2}=\sigma_{1}^{2}$ (Y does not cause X ) and H_1_: $\sigma_{2}^{2}<\sigma_{1}^{2}$(upper tail , Y causes X)
4. If the F-test rejects the null Hypothesis at the .01 significance level then Y🡪X
5. Similar procedure for testing X🡪Y
6. This procedure was followed for all subjects, for left-to-right and right-to-left foot acceleration MM spike series causalities and in all cases we found that the null hypothesis was rejected ( << 0.01)
